# Impact of educational interventions provided to patients with a central venous catheter and their informal caregivers: a systematic review

**DOI:** 10.1186/s13756-025-01583-w

**Published:** 2025-06-11

**Authors:** L. Foucault-Fruchard, N. van der Mee, C. Reinprecht, C. Durand, J. Cailhol, J. R. Zahar, S. Kerneis

**Affiliations:** 1 CHU de Tours, 2 Boulevard Tonnellé, Tours, 37000 France; 2 IAME, Université Paris Cité, 16 Rue Henri Huchard, Paris, 75018 France; 3 Equipe « Bactéries et Risque Materno-fœtal », Unité Mixte de Recherche (UMR) 1282, Infectiologie et Santé Publique INRAE, 10 Boulevard Tonnellé, Tours, 37 000 France; 4 Equipe de Prévention du Risque Infectieux, Hôpital Bichat, APHP, 46 Rue Henri Huchard, Paris, 75018 France; 5 Laboratoire Éducations et Pratiques de Santé, Université Sorbonne Paris Nord, 74 Rue Marcel Cachin, Bobigny, 93017 France; 6 Service Maladies Infectieuses, Groupe Hospitalo-Universitaire Paris Seine Saint Denis, Assistance Publique des Hôpitaux de Paris, 125 Rue de Stalingrad, Bobigny, 93000 France; 7 Unité de Prévention du Risque Infectieux, Groupe Hospitalo-Universitaire Paris Seine Saint Denis, Assistance Publique des Hôpitaux de Paris, 125 Rue de Stalingrad, Bobigny, 93000 France; 8 Pharmacie À Usage Intérieur (PUI), Hôpital Bretonneau – CHU de Tours, 2 Boulevard Tonnellé, Tours, 37000 France

**Keywords:** Educational interventions, Therapeutic education, Central venous catheter, Patients, Informal caregivers, Complications, Infections

## Abstract

**Background:**

Central venous catheters offer considerable benefits, but their presence can expose patients to serious complications. Preventing such complications is crucial, not only for individual patients, but also for hospitals and the healthcare system.

**Objective:**

To assess the impact of educational interventions on clinical and non-clinical outcomes provided to patients with central venous catheters and/or their informal caregivers, regardless of the therapeutic indication, and to define the specific characteristics of effective educational strategies.

**Methods:**

Medline and Embase were searched, covering all publications since their inception to 13 August 2024. Articles on clinical and/or non-clinical outcomes related to the education of patients with central venous catheters and their informal caregivers were included. Studies focusing solely on the education of healthcare providers were excluded. The reference lists of included studies were hand searched for additional citations. This systematic review followed PRISMA guidelines and the protocol was registered in PROSPERO (CRD42024577193). The quality of the included studies was assessed using the Mixed Methods Appraisal Tool.

**Results:**

In total, 20 articles, representing 974 patients and 875 informal caregivers, were included in the review: seven randomized trials, ten quantitative studies without randomization, and three descriptive quantitative studies. The compliance rate of 60% for the randomised trials determined using the Mixed Methods Appraisal Tool indicates a low risk of bias, whereas non-randomised and descriptive quantitative studies show more methodological weaknesses (40% and 45%, respectively). There was a positive trend, significant or not, for the impact of patient education on reducing complications, particularly those related to infection (85% of the studies concerned by this outcome). This was often observed (64%) in studies based on educational interventions repeated over time. Studies that showed a significant improvement in patients’ knowledge and skills in terms of self-management showed beneficial results in terms of the occurrence of complications. Nurses were the most common educators (15/20), and the most frequently used tools were written materials and digital resources, often combined with other methods for greater effectiveness.

**Conclusions:**

This systematic review encourages the implementation of educational interventions for patients with central venous catheters and their informal caregivers, notably to decrease infections. Providing them with written documents and digital tools, and delivering them repeatedly over time, should be promoted. However, study heterogeneity limits definitive conclusions. Future research should standardize methodologies, involve patients in intervention design, and assess cost-effectiveness to ensure sustainable implementation.

**Supplementary Information:**

The online version contains supplementary material available at 10.1186/s13756-025-01583-w.

## Introduction

Central venous catheters (CVCs) include peripherally inserted central catheters (PICCs), tunnelled or non-tunnelled central catheters, and fully implanted ports (PORTs). CVCs offer a number of important advantages, including long-term placement, easy blood sampling without repeated venipuncture, and the safe administration of drugs and irritant fluids. The increasing incidence of chronic illnesses and the growth of outpatient medicine have led to the increasing use of these devices throughout the world.

However, the presence of a CVC expose patients to serious complications, including infection, thromboembolism, and mechanical complications [1–4]. Catheter-related infections (CRIs) and deep vein thrombosis can lead to longer hospital stays, increased healthcare costs, and a higher risk of mortality [1, 5–10]. The incidence of central line-associated bloodstream infection (CLABSI) varies depending on the clinical context, but most published studies report an incidence of between 0.5 and 10 per 1,000 catheter days [11–14]. These complications represent a major financial burden for healthcare systems, with CLABSIs alone contributing to billions of dollars in annual costs [8, 15]. Thus, effective preventing strategies are critical to improving patient safety and reducing healthcare costs.

Historically, the prevention of CRIs has been largely the responsibility of healthcare professionals, with a strong emphasis on intensive care units (ICUs), where infection control guidelines have prioritized adherence to best practice [16, 17]. Over time, this approach has been extended to other healthcare settings beyond the ICU, particularly onco-haematology units [18, 19]. However, patients and their informal caregivers also have a crucial role to play in infection prevention. Involving patients in safety initiatives can improve compliance with hygiene measures [20]. Despite this, many patients report inadequate education about the CVC-related risks. In a study of Anderson et al., 40% of patients expressed a need for better education about the risks associated with central venous access [21]. This highlights a critical gap in patient education that may contribute to suboptimal adherence to preventive measures. Patient education consists of concrete patient training, which leads patients and their support network to develop skills in self-care and disease adaptation, as defined by the World Health Organization [22].

Despite increasing recognition of education of patient with CVCs and/or informal caregivers as a preventive strategy, the optimal approach remains unclear. Existing educational interventions vary widely in content, format, and frequency, making it difficult to determine which strategies are the most effective. Additionally, there is a lack of comprehensive evaluation of their clinical and non-clinical effects.

In this context, the aim of this systematic review was to assess the impact of educational interventions on clinical and non-clinical outcomes provided to patients with CVCs and/or their informal caregivers, regardless of the therapeutic indication, and to define the specific characteristics of effective educational strategies.

## Methods

This systematic review was performed according to Preferred Reporting Items for Systematic reviews and Meta-Analyses (PRISMA) guidelines [23]. The review protocol was registered in the International Prospective Register of Systematic Reviews (PROSPERO) with the number CRD42024577193.

### Data sources and search strategy

A literature search of the databases MEDLINE and EMBASE was conducted on 13 August 2024, including all relevant articles published from the inception of each database until the search date. The search strategy was developed by the authors in consultation with two academic librarians. The following search strategy was used in MEDLINE: "(((education intervention [Title/Abstract]) OR (patient education [Title/Abstract]) OR (intervention program [Title/Abstract]) OR (education program [Title/Abstract])) AND (catheter [Title/Abstract]))". This search strategy was adapted to the syntax of EMBASE.

### Inclusion criteria

French- and English-language studies that reported original research were considered. To be eligible for inclusion, a study had to report clinical and/or non-clinical outcomes related to the education of patients with a CVC (of any type), all types combined, and/or their informal caregivers. The studies included in the review covered the education of patients who had been hospitalized and then transferred to outpatient care, as well as those who were managed in hospital only or outpatient care only. Given the reasonable number of studies included, all clinical and non-clinical outcomes, regardless of their type or apparent importance, were rigorously considered to ensure a complete assessment of the effects observed. No restrictions were imposed on the study design.

### Exclusion criteria

Studies that focused solely on the education of healthcare caregivers (i.e., physicians or nurses) were excluded. Case reports, literature reviews, conference proceedings, and abstract were also excluded.

### Data extraction and analysis

**Fig. 1:**
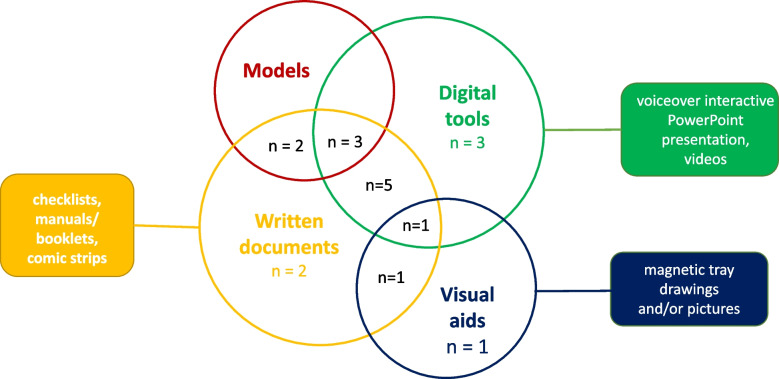
Teaching tools used in the studies analysed. The numbers in the circles indicate the number of studies concerned

Rayyan software was used for sorting the articles, the management of duplicates, and screening. First, duplicate articles were identified and removed from the database records. Then, titles and abstracts were independently screened based on the inclusion criteria by two authors (LFF and JRZ). Discrepancies were resolved by discussion with reference to the protocol criteria. If a consensus could not be reached, a third reviewer was available for discussion (SK). Subsequently, the full text of articles that met the inclusion criteria was extracted and independently analysed by two authors (LFF and JRZ) (Fig. 1).

A standardized electronic data-charting form was created using Microsoft Excel® software to collect the following data from the included studies: first author, publication date, name of the journal, country, type of medical device concerned, data design, study aims, study population, and sample size. Data concerning the educational intervention, outcome measures, and key results related to the objective of this review were also collected in the structured form. The reference lists of included studies were hand searched for additional citations.

### Data synthesis

A systematic narrative synthesis was conducted to summarize and compare the impact of educational interventions on clinical and non-clinical outcomes, and their specific characteristics. Studies were grouped according to the type of educational tools used and reported outcomes. Key similarities and differences between studies were analysed, taking into account variations in intervention content and outcome assessment methods. A meta-analysis was not performed because of the heterogeneity of the included studies.

### Quality assessment

The quality and risk of bias of each included study were assessed using the Mixed Methods Appraisal Tool (MMAT) [24]. The assessment was conducted independently by two reviewers (LFF and JRZ). The disagreement rate between the two evaluators was low. For cases of divergence, the evaluators conducted a joint review of the articles to reach a consensus.

## Results

### Results of the search

The search strategy produced 543 results after removing duplicates (Fig. 1 in the Supplement). In total, 528 were excluded based on the title and/or abstract. The full text of the remaining 15 articles was screened for eligibility, of which 14 were selected. Six additional articles were found through hand-searching. Finally, 20 articles were included in the review.

### Characteristics of the included studies

The main characteristics of the included studies are listed in Table 1. Among the 20 studies, most were performed in North America (*n* = 8), followed by Asia (*n* = 5), Europe (*n* = 5), Australia (*n* = 1), and South America (*n* = 1). The publication dates ranged from 2003 to 2024. Seven studies included patients with PICC Lines [25–31], eight with tunnelled CVCs [31–38], and six with an unspecified type of CVC [39–44]. In total, 974 patients and 875 caregivers were included in this review. Most studies were randomised controlled trials [26, 29, 33, 35, 25, 36, 39] or quasi-experimental [28, 30–32, 37, 40, 41, 44]. In four studies, the control arm did not consist of patients naive to an educational intervention [29, 35, 36, 40]. In these studies, standard educational interventions were compared with standard education combined with new technology (i.e., videos or a voiceover interactive PowerPoint presentation).

**Table 1:** Characteristics of the studies, population, and educational intervention in the studies analysed

**Author(s) (year)** **Country** **[Ref. No.]**	**Characteristics of the studies**	**Characteristics of the population**			**Characteristics of the intervention**
	**Study design, sample size**	**Age of educated group/context of central venous catheter placement**	**Type of CVC**	**Catheterization time**	
De La Maza et al. [43] Chile	Prospective, non-randomised, experimental study. CG: 50, EG: 52	Adults (the parents or legal guardians of children)/Chemotherapy	NS	NS	Nursing educators. Face-to-face individual sessions, 4 h in total over 3 consecutive days. Caregivers received a printed copy of the content
Drews et al. [42] USA	Retrospective comparative study CG: 45, EG: 80	Adults (caregivers)/Parenteral nutrition	NS	NS	Nursing educators. Face-to-face interviews. Use of a video and a competency checklist of CVC care skills. Educational intervention over 4 days with 1 session/day
Emery et al. [35] USA	Prospective, randomized, controlled pilot study CG: 27, EG:24	Adults/Parenteral nutrition	Tunnelled catheter (Hickman) PICC line	*Mean* ± *SD*: CG 2.1 months, EG 2.4 months	Nursing educators. Patient education included 2–3 didactic lessons. They also received a manual on catheter care and a televised video on home self-monitoring. Learning of technical gestures on a model
Hicks et al. [44] USA	Quasi-experimental research (pre-post intervention). *n* = 105	Adults (caregivers)/NS	NS	NS	Nursing educators. Collective sessions. Multimodal methods, including teach-back, demonstration, short videos, handbook with clear content and photos, hands-on-practice and verbal explanation
Hilberath et al. [38] Germany	Retrospective study, pre-post intervention. *n* = 117	Adults (caregivers)/Parenteral nutrition	Tunnelled catheter	*Total days:* 248 864	Educational program with 7–10 sessions (one session/day). Both theoretical knowledge and practical skills (on manikins). Written educational materials were provided to patients and caregivers
Li et al. [29] China	Prospective randomized controlled trial CG: 63, EG: 66	Adults/Chemotherapy and parenteral nutrition	PICC Line	NS	Nursing educators. Individualized education. Monthly meetings to watch and discuss home care videos of PICC patients. An education manual is given to patients
Liu et al. [26] China	Two-parallel randomized controlled trial CG: 80, EG: 79	Adults/Chemotherapy	PICC Line	CG: ≤ 30 days *n* = 26, > 30 days *n* = 54 EG: ≤ 30 days *n* = 30, > 30 days *n* = 49	Nursing educators. Individualized education to teach PICC maintenance and self-management techniques. Monthly meetings to watch and discuss home care videos of PICC patients
Lo vecchio et al. [37] Italy	Quasi-experimental research *n* = 120	Adults (caregivers)/Chemotherapy and other medication	Hickman—Broviac and Groshong Catheters	NS	In total, 9 educational sessions. Both theoretical knowledge and practical skills (on mannequins/patients). A video and a checklist for CVC management at home was distributed to participants
Moller et al. [33] Denmark	Prospective, randomized, controlled intervention study. CG: 40, EG: 42	Adults/Chemotherapy	Tunnelled catheter	*Total days:* CG 710, EG 6264	Nursing educators. Individualised patient education. Both theoretical knowledge and practical skills. The educational programme started 1 to 6 weeks after catheter insertion
Moller et al. [34] Denmark	Observational study *n* = 60	Adults/Chemotherapy	Tunnelled catheter	NS	Nursing educators. Meeting with the individual patient and his/her spouse for 1 h. Patients received written documentation, a thermometer, and a patient diary
Park et al. [32] South Korea	Quasi-experimental, sequential cohort design study. CG: 24, EG: 21	Adults/Chemotherapy	Tunnelled catheter	*Total days:* CG 1841, EG 1603	Nursing educators. Both theoretical knowledge and practical skills. (on models). Patients receive a CVC management booklet. Evaluation of CVC self-management tasks using a checklist. Educational intervention every week over 6 weeks
Petroulias et al. [30] USA	Quasi-experimental research *n* = 11	Adults/Chemotherapy	PICC Line	NS	Nursing educators. Face-to-face session nurse and coaching via FaceTime for their first independent flushing procedure. Access to a video that outlined the 10 steps of flushing
Pierick et al. [40] Canada	Quasi-experimental research (before/after study) *n* = 11	Adults (caregivers)/Parenteral nutrition	NS	NS	Nursing educators. Individual training program with printed materials (manual). Families and caregivers were involved in the production of 3-to-5 min educational videos
Raybin et al. [36] USA	Randomized controlled clinical trial CG: 21, EG: 33	Adults (caregivers) and paediatrics/Chemotherapy and other medication	Tunnelled catheter	NS	Nursing educators. A face-to-face education (60 min) and use of a DVD (20 min) to watch at home
Smith et al. [39] USA	Randomized placebo-controlled clinical trial CG: 38, EG: 35	Adults/Parenteral nutrition	NS	NS	Nursing educators. Each educational intervention packet included a videotape (12 to 15 min) with accompanying pamphlet, a self-monitoring check-list, and a diary
Tan et al. [41] Taiwan	Pretest − posttest two-group quasi-experimental study design. CG: 30, EG:30	Adults (patients and caregivers)/Surgery, administering fluids and nutrition	NS	*Mean* ± *SD*: CG 8.25 ± 3.68 days*;* EG 8.37 ± 5.09 days	Face-to-face individual training sessions. Patients received an instructional handbook, a video corresponding with the contents of the handbook, and a checklist of CLABSI prevention guidelines
Veyrier et al. [27] France	Observational study *n* = 30	Adults/Chemotherapy and antibiotherapy	PICC Line	*Total days:* 1659 *Mean* ± *SD*: 42 days ± 29; median: 37 days	Pharmacist educators. Two face-to-face individual interviews during hospitalization. Use magnetic tray and illustrated cards. Patients received a comic strip based on the above images
Wang et al. [25] China	Prospective, randomized, controlled study CG: 60, EG: 60	Adults/Chemotherapy	PICC Line	CG: 14–30 days *n* = 20, 31–90 days *n* = 27, > 90 *n* = 13 EG: 14–30 days *n* = 23, 31–90 days *n* = 23, > 90 days *n* = 14	Previous patients were invited to give lectures. Patient education cards and brochures were distributed. The complications were explained using WeChat groups and telephone follow-up. Educational videos on the maintenance of PICCs and checklists were used to supervise the patients and their families to independently carry out daily catheter maintenance
Wong et al*. *[31] USA	Quasi-experimental research *n* = 181	Adults (caregivers) ± paediatrics (optional participation)/NS	PICC Line Tunnelled catheter	NS	Nursing educators. Pre-discharge education on CVC care: watched videos; observation of CVC care either on their child or through group classes; multiple hands-on-practice and teach-back, until independent. Caregivers received a practical guide. After hospital discharge, they participated in the clinical teach-back program (demonstration on patients or models). Every 90 days if CVC still in place or patient experienced a CLASBI: teach-back refresher
Yap et al*. *[28] Australia	Quasi-experimental research, retrospective-prospective comparative study. CG: 27, EG: 73	Adults/Chemotherapy, administering fluids and medication	PICC Line	*Total days:* 6872 *Mean*: 78.1 days*; Median:* 44 days (1–524 days)	Nursing educators. No information on the teaching tools used

*CG* control group, *CLASBI* Central Line-associated Bloodstream Infection, *CVC* Central Venous Catheter, *EG* experimental group, *NS* not specified, *PICC* Peripherally Inserted Central Catheter

### Ratings of the quality of the evidence

The compliance rate of the randomized trials with the MMAT criteria was 62%, indicating a low risk of bias. In contrast, quantitative studies without randomization and descriptive quantitative studies showed more methodological weaknesses. Their compliance rates were 44% and 55%, respectively. These results are partially explained by the lack of data on the reasons why certain eligible individuals chose not to participate and any attempts to obtain a sample of participants that represented the target population. Overall, only two studies achieved an MMAT score ≥ 4, while the remaining studies had low to very low scores (ranging from 1 to 3) [33, 39]. The MMAT quality assessment results are presented in Table 2. For more details, please refer to Table 1 in the Supplement.

**Table 2:** Scores for assessing the quality of studies using the MMAT (Mixed Methods Appraisal Tool)

	**Overall score**
Randomized trials	
Emery et al*. *[35]	2/5 (40%)
Li et al*. *[29]	3/5 (60%)
Liu et al*. *[26]	3/5 (60%)
Moller et al*. *[33]	5/5 (100%)
Raybin et al*. *[36]	2/5 (40%)
Smith et al*. *[39]	4/5 (80%)
Wang et al*. *[25]	3/5 (60%)
Average score	3.1/5 (62%)
Quantitative studies without randomization	
De la Maza et al*. *[43]	3/5 (60%)
Drews et al*. *[42]	2/5 (40%)
Hicks et al*. *[44]	2/5 (40%)
Hilberath et al*. *[38]	3/5 (60%)
Lo Vecchio et al*. *[37]	3/5 (60%)
Park et al*. *[32]	2/5 (40%)
Pierick et al*. *[40]	1/5 (20%)
Tan et al*. *[41]	2/5 (40%)
Wong et al*. *[31]	2/5 (40%)
Yap et al*. *[28]	2/5 (40%)
Average score	2.2/5 (44%)
Descriptive quantitative studies	
Moller et al*. *[34]	3/4 (75%)
Petroulias et al*. *[30]	2/5 (40%)
Veyrier et al*. *[27]	2/4 (50%)
Average score	2.3/5 (55%)

The studies were classified according to type and evaluated on five specific criteria. The overall score represents the number of criteria met out of the five assessed

### Characteristics of the study populations

Educational interventions mainly concerned only adults as patients or informal caregivers. Only one study targeted both children and their informal caregivers [36]. In a study by Wong et al., children’s participation alongside caregivers was not compulsory but encouraged [31]. CVCs were indicated for chemotherapy (*n* = 12) [25–30, 32–34, 36, 37, 43], parenteral nutrition (*n* = 7) [29, 35, 38–42], or another reason (IV fluids/medications) (*n* = 4) [27, 28, 36, 41]. This variety of indications led to a very heterogeneous duration of catheter placement between studies, ranging from a few days [41] to several months [31, 33]. The level of health literacy of the participants was not assessed in the included studies. However, some authors specified the participants’ level of education [25, 26, 29, 30, 32, 39, 41, 44]. The main characteristics of the study populations are listed in Table 1.

### Description of the educational approaches evaluated in the included studies

Nurses were the healthcare professionals most frequently represented as educators [26, 28–36, 39, 42–44]. Pharmacists were involved in one study [27]. In the other studies, the profession of the educator was not specified. In five studies, patient education was integrated with training for healthcare professionals on the insertion, use, maintenance, and monitoring of CVCs [28, 31, 37, 38, 42].

A wide range of pedagogical tools was used (Fig. 1). Written documents and digital tools were the tools most commonly used to transfer knowledge and skills to patients. Written documents were provided in booklets/manuals [29, 31, 32, 34, 35, 38–41, 43, 44], comic strips [27], and/or checklists [25, 32, 37, 39, 41]. In 85% of cases, written documents were combined with one or more additional teaching tools: models [31, 32, 35, 37, 38], digital tools [25, 29, 31, 35, 37, 39–41, 44], and/or visual aids [25, 27]. Digital tools mainly consisted of videos [25, 26, 29–31, 35–37, 39–42, 44]. As for written documents, digital tools were frequently integrated with another teaching tool. In one study, the complications were explained to the patients using patient education through WeChat groups and telephone follow-up [25]. Among the visual aids, educational teams opted for pictures/drawings [25, 27] or a magnetic tray.

In terms of teaching methods, meetings or face-to-face interviews were predominant [26, 27, 29, 30, 34, 36, 37, 41, 43]. Some educators relied on sharing experiences or providing feedback [32, 40]. In the study of Wang et al., previous patients who had been successfully treated were invited to give lectures [25]. Concerning videos, patients and/or caregivers frequently watched them independently, whether during hospitalization or at home. Viewing of the video could be followed by a discussion with a healthcare professional to address any questions [29]. In eight studies, acquisition of the required skills relied on performing technical procedures under the instructor’s supervision [30–33, 35, 37, 38, 42]. Technical procedures could be practiced on a model or directly on the patient. In one study, supervision was conducted remotely via video [30].

### Effect of educational interventions on outcomes related to patients with central venous lines

**Fig. 2:**
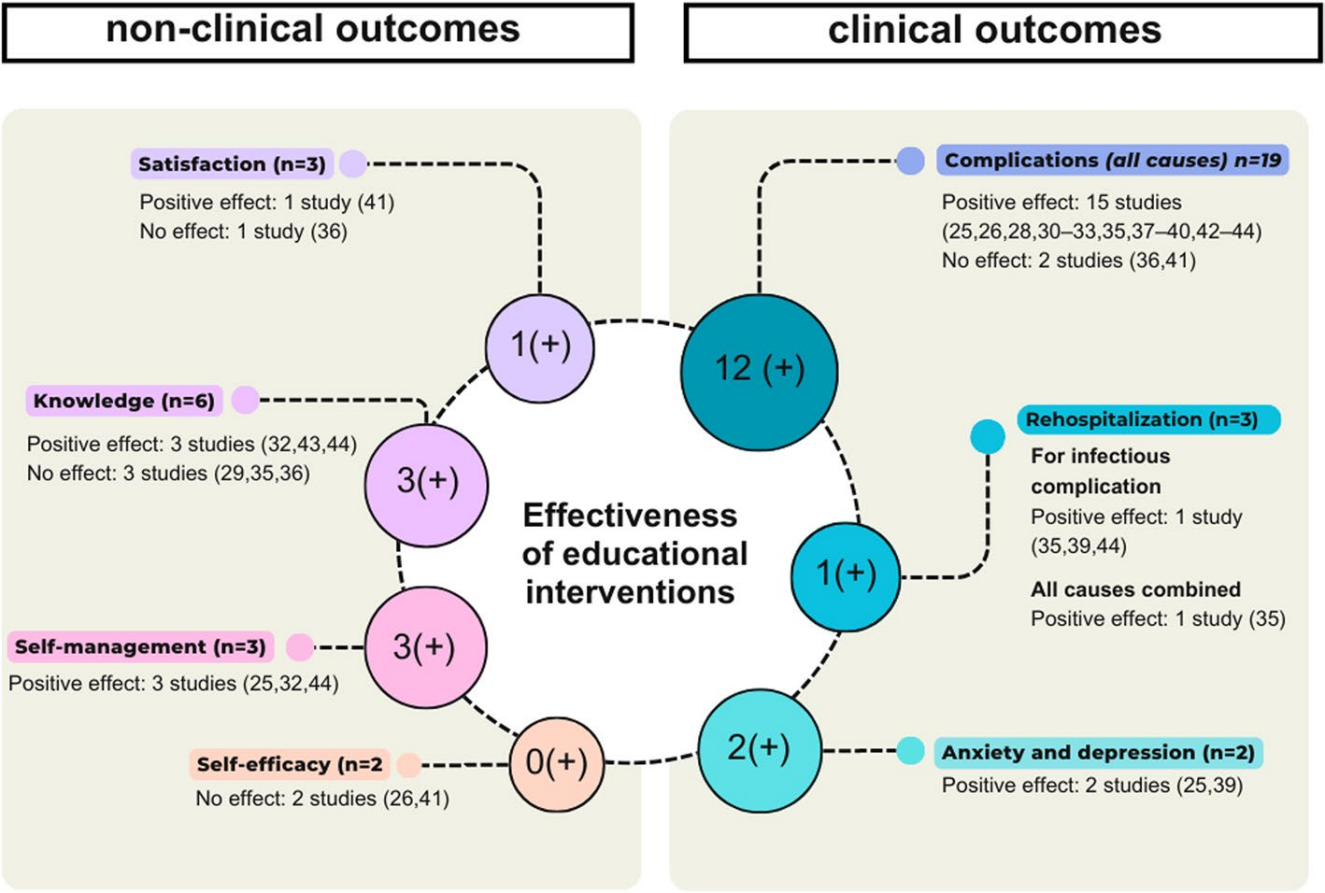
Effectiveness of educational interventions categorized by the outcomes studied. The numbers in the circles indicate the studies with significant positive results for each outcome. Only studies with statistical analysis of the outcomes are shown in this figure

The main outcome measured was the occurrence of infectious, thrombotic or mechanical complications (e.g., displacement, rupture, obstruction) [25–28, 30–43] (Fig. 2). A significant positive effect was observed in 12 of the 19 studies focusing on these criteria [26, 28, 30, 32, 33, 37–40, 42, 43, 25]. It is worth noting that, two of these studies have a very high level of quality according to the MMAT score [33, 39] while five studies are of low to very low quality [28, 30, 32, 40, 42]. Moreover, this significant beneficial effect on the occurrence of complications was demonstrated in two-thirds of the randomized controlled trials [25, 26, 33, 39]. Notably, the three quarters of them were associated with a positive effect on the occurrence of infectious complications. Two studies that focused on complications as outcomes did not have a comparison group [27, 34].

**Fig. 3:**
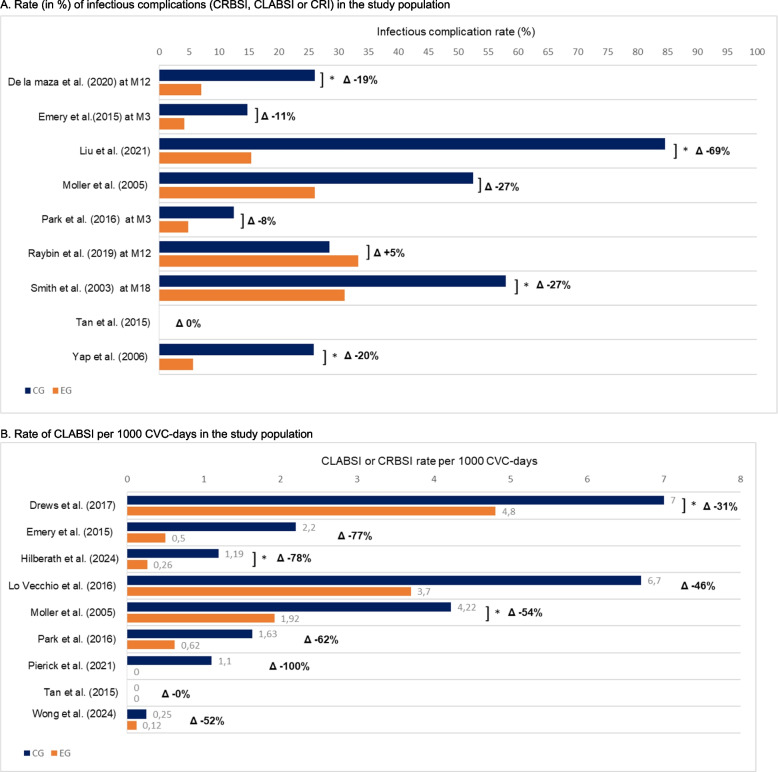
Infectious complications in the studies analysed. **a** Rate (in %) of infectious complications (CLABSIs, CRBSIs or CRI) in the study population. An asterisk (*) indicates that a significant difference was observed between the control group (CG, in blue) and the experimental group (EG, in orange). The differences observed between the two groups (Δ in %) is mentioned at the end of each horizontal bar. The time of reporting of infectious complications is indicated next to the authors’ names, when this information was available. CLABSI: central line-associated bloodstream infection, CRBSI: catheter-related bloodstream infection, CRI: catheter related infection, M: time elapsed in months between inclusion of the patient in the study and reporting of an infectious complication. **b** Rate of CLASBI or CRBSI per 1000 CVC-days in the study population. An asterisk (*) indicates that a significant difference was observed between the control group (CG, in blue) and the experimental group (EG, in orange). The differences observed between the two groups (Δ in %) is mentioned at the end of each horizontal bar. CLABSI: central line-associated bloodstream infection, CRBSI: catheter-related bloodstream infection, CVC: central venous catheter

Regarding infectious complications, most studies focused on CLABSIs or CRBSIs [31–33, 35–42]. However, the exact definition used for diagnosis was rarely reported [26, 43]. When the authors did, they did not always explicitly refer to the Centers for Disease Control and Prevention (CDC) criteria. Overall, the definitions found in these studies were generally consistent with the CDC definition, which describes CLABSI as a primary bloodstream infection that occurs in a patient with a CVC in place within the 48 h before the onset of infection, with no other identifiable source of infection. According to the CDC, unlike CLABSI, CRBSIs require strict microbiological criteria confirming the catheter as the source of infection. In our review, the term CRIs covers infections in a broad sense, including both CLABSIs or CRBSIs and local infections. In particular, the study by Mollet et al. provided a precise definition of local exit-site infection [33]. Overall, the rate of CLABSIs, CRBSIs or CRIs was significantly reduced in four studies [26, 28, 39, 43] (Fig. 3A). However, in hospitals, the incidence rate is a key indicator used to monitor and improve the quality of care. It is expressed as the number of new CLASBIs [31, 37, 38, 40–42] or CRBSIs [32, 33, 35] occurring in a given population per 1000 central line days used. Of these nine studies, only three showed a significant reduction between the experimental and control groups [33, 38, 42] (Fig. 3B).

In contrast, the occurrence of non-infectious complications is more anecdotal in the studies included in this review. All studies that focused on overall complications, including infectious or non-infectious complications (e.g., occlusion, displacement, line break, phlebitis, bleeding), reported a significant reduction in their overall occurrence as a result of educational interventions [25, 26, 28, 32, 40]. Only one study specifically highlighted a significant reduction for a particular type of non-infectious complication [26]. Indeed, according to Liu et al., displacement occurred in 83.3% of patients in the control group experienced displacement compared with 16.67% in the educational group (*p* = 0.016) (Table 2 in the Supplementary Material 3).

In addition, only three studies have assessed the impact of educational interventions on rehospitalization rates due to CRBSIs or from all causes combined (including reasons unrelated to complications, such as weight variations) [35, 39, 44]. While Smith et al. showed a significant positive effect of the educational intervention on the rate of rehospitalization due to infectious complications [39], Emery’s study found no significant effect of the intervention on the overall rehospitalization rate [35]. The authors also examined the impact of educational interventions on self-efficacy and self-management. While self-management focuses on practical strategies and actions to manage one’s life effectively, self-efficacy focuses on the belief in one’s own ability to succeed. Favourable results were observed in the three studies that investigated self-management [25, 32, 44]. The studies of Park et al. and Hick et al. involved a questionnaire developed by the authors to evaluate the outcome, whereas the study of Wang et al. used the seven-dimensional Cancer Patients PICC Self-management Scale (CPPSM). Conversely, no effect was observed in the studies that assessed self-efficacy using validated scales, i.e., the General Self-Efficacy Scale (GSES) and Caregiver Self-Efficacy Scores (CSESs) [26, 41]. The effects observed on patients’ level of knowledge [29, 32, 35, 36, 43, 44] and satisfaction [27, 36, 41] varied widely between studies. Because no valid and reliable instruments are available, knowledge was evaluated using questionnaires developed by the authors. Finally, positive effects were observed for anxiety and depression [25, 39], the number of calls to clinicians by patients [35], and of problem-solving with professionals [39] (Table 2 in the Supplementary Material 3).

## Discussion

### Interpretation of the results

Overall, educational interventions seem to have a more pronounced positive effect on clinical outcomes, measured in almost 90% of the articles, especially on the reduction of complications. This effect was highlighted in two high-quality studies according to the MMAT score [33, 39] and in two-thirds of randomised controlled trials [25, 26, 33, 39]. The most commonly assessed type of complication is infectious complications, with a comparable trend being observed in several studies, showing significant reductions up to 100% [40]. This trend suggests that patient and/or informal caregiver education may play an important role in infection prevention, possibly by improving adherence to hygiene measures and early recognition of infection symptoms. In addition, some studies show no significant effect, highlighting the variability in the impact of educational interventions depending on the study population and the strategies used, including the content, delivery method and duration of the educational intervention. Similar results were reported by Genova Vieira et al. [45]*.* Their systematic review evaluated the effectiveness of teaching–learning programs exclusively for cancer patients and/or their caregivers or family solely in preventing and controlling infections.

For rehospitalization, the effect of educational interventions was more nuanced. While a positive effect is observed in all studies, there is no significant evidence of an effect on overall rehospitalization. This may indicate that although education helps to prevent certain specific types of complications, it does not have a general effect on reducing hospital admissions, possibly because of other influencing factors (such as comorbidities). In terms of mental health, educational interventions seem to have a beneficial effect, with two studies reporting improvements in anxiety and depression. This may be explained by an increased sense of control in patients who benefit from these interventions.

From a non-clinical perspective, the effects of educational interventions were more heterogeneous. While knowledge acquisition shows mixed results, self-efficacy does not seem to improve. This discrepancy may suggest that knowledge acquisition alone is not sufficient to increase patients’ confidence in managing their health. It is possible that other factors, such as the way information is delivered, the level of patient engagement and the presence of ongoing support, play a crucial role in translating knowledge into perceived competence.

In contrast, several studies showed a significant improvement in patients’ level of knowledge and self-management, with more positive clinical outcomes observed for infections [32, 43, 44]. This trend was less pronounced in terms of impact on rehospitalization rates due to infectious complication or all causes [44].

Therefore, our review highlighted the overall effectiveness of educational interventions, particularly in reducing clinical complications and improving certain psychological aspects. However, the conflicting results observed in the studies included in our review may be due to several factors. First, the heterogeneity of study designs and outcome measures makes direct comparisons difficult. Variability in the timing of outcome assessment in relation to CVC placement and in the duration and frequency of educational interventions may also influence their effectiveness. In addition, some trials evaluated patient education alongside other health care interventions, which may overestimate its independent effect. These inconsistencies highlight the need for more standardised methods and high-quality studies to clarify the true effectiveness of patient education on clinical outcomes, particularly complications.

### Characteristics of educational methods

Educational interventions repeated over time, weekly for 1.5 to 2 months or monthly for the entire duration of CVC placement, were more often associated with a reduction in the risk of complications [26, 32, 33, 37, 42]. Emery et al. suggested that too much teaching over a short period of time may not improve knowledge. On the contrary, it may diminish students’ attention and concentration on the education [35]. Given the natural risk of knowledge loss, it would be informative to evaluate the impact of an educational intervention after a certain period of time from its implementation. Only two studies evaluated the rate of infection at 6, 12, and 18 months [39, 43]. While caregivers’ involvement is essential for optimal treatment, notably of cancer patients, only a quarter of the studies concerned informal caregivers in oncology [36, 37, 43].

Most of the studies mentioned the use of written documents, often combined with digital tools, such as videos or voiceover interactive PowerPoint presentations. Digital tools have developed significantly over the last 10 years. According to Li et al., they have advantages in terms of educational practices (distance learning possible) and saving time for educators [29]. This argument, combined with the possibility of creating video content using a smartphone, has a beneficial economic impact [40]. Although the rate of video viewing by patients differed between studies [35, 40], the prevalence of positive outcomes suggests that the use of video is effective. Similar results have been observed for educational approaches interventions related to various medical conditions, such as surgery [46]. However, adding educational videos to a pre-existing educational approach did not significantly reduce infectious complications [35, 40]. In addition, certain educational interventions aimed to help patients acquire technical skills, such as flushing a catheter [30, 36, 40, 42], mainly using digital tools. Although patient education is not a one-sided process of transforming information, simulation-based learning to acquire technical skills using a model or manikin was rarely found in the studies analysed. However, this is an effective teaching strategy to enable patients to actively contribute to their own care [47]. Of note, few studies specified whether the content of the educational interventions was co-constructed with patients and/or caregivers. Involving patients as stakeholders in the development of educational interventions is essential to meet their expectations and adapt the messages conveyed [48]. Such patient involvement in the process enabled Veyrier et al. to revise the information communicated during face-to-face interviews, some of which the patients found highly anxiety-provoking [27].

### Limitations of the evidence included in the review

More research using high-quality study designs is needed to fully objectify the possible potential of patient education on infectious complications, as suggested by Genova Vieira et al*.* [45]. Only seven studies were randomised controlled trials. Also, three technical issues emerged from this review, which needs to be addressed to allow comparability between studies. The first is to systematically mention the time elapsed between implementation of the educational intervention and the outcomes notification. The second is to harmonise the indicator used to measure infection incidence, for instance by using the incidence rate per 1000 days of catheterisation. Organisations, such as the WHO and the CDC, have established guidelines for the surveillance and prevention of catheter-associated infections, confirming the use of incidence rate per 1000 catheter days as an internationally recognised quality indicator. This indicator enables a fair comparison to be made between healthcare establishments of different sizes or with varying levels of activity. The third is to define CRI, CRBSI or CLABSI, which serve as the basis for diagnosis. These definitions were not systematically specified in the included studies, which makes comparisons difficult. In addition, patient knowledge was assessed based on the number of correct answers to a questionnaire developed by the authors. Using a published validated questionnaire to assess patient knowledge provides advantages in terms of reliability, validity, and comparability, thus contributing to more accurate assessments of patient knowledge. Furthermore, some of the studies incorporated the combined impact of patient education along with that of medical and paramedical caregivers in their evaluation [28, 31, 37, 38, 42]. This may have led to an overestimation of the results observed and attributed to patient education. Finally, we did not perform a regression analysis because of the limited number of studies available for each outcome, which restricted the possibility of identifying robust associations. Lastly, the overall statistical power of the studies was limited, mainly because of small sample sizes. These limitations should be taken into account when interpreting the results.

### Limitations of the review processes used

The review process we used had several limitations. First, we only included articles written in English or French. Thus, there was a linguistic and cultural bias. Moreover, the literature search was limited to two databases (MEDLINE and EMBASE). Although these are important sources for medical research, this limitation may have reduced the comprehensiveness of the analysis and increased the risk of missing relevant studies. Furthermore, conference proceedings were not included because they contained very little detail about the educational intervention, which may have led to selection bias. As with any journal, publication bias is also likely. This can lead to an over-representation of interventions with positive results in the literature, thus distorting the conclusions of our systematic review. Finally, the strong heterogeneity of the interventions and outcomes prevented us from carrying out a quantitative synthesis (meta-analysis).

### Implications of the results for practice and future research

The low quality and limited evidence of the studies included in this review provide insufficient evidence to determine with certainty which interventions are most effective, for whom and in which context. However, the interventions that merit further study are educational interventions that rely on several educational tools, including digital tools and written documents, and that are repeated over time. Indeed, recently published systematic reviews and meta-analyses encourage the use of digital tools in patient education such as patients with diabetes or heart failure [49–51]. Given the encouraging results of studies combining patients and caregivers [28, 42], it would be worthwhile to pursue such efforts in future research and to develop the acquisition of technical skills through simulation. This would make it possible to identify standardized educational tools, as they are currently lacking for the education of patients with a CVC [36]. To improve comparability, the incidence density of CLABSIs should be assessed systematically, and a standardised, validated questionnaire should be used to assess knowledge. Healthcare professionals must ensure that patients and their caregivers understand information about the self-management of CVCs by providing clear and ongoing health literacy education. Thus, it would be informative to analyse outcomes in greater depth according to patient characteristics (for example, the level of health literacy or socio-economic level) to determine the most effective interventions in relation to the socio-demographic characteristics of patients. Future research should systematically be coupled with medico-economic studies, as their results would support the sustainability of established programs and encourage the implementation of educational interventions in healthcare establishments that do not currently offer such services to patients.

## Conclusion

The results of this systematic review suggest that educational interventions for patients with CVCs and their informal caregivers could be beneficial, notably in reducing infectious complications. The evidence suggests that combining written materials with digital tools could be an effective strategy, especially when interventions are delivered repeatedly over time. However, the heterogeneity of study designs, outcome measures, and educational methods limits the ability to draw definitive conclusions. While some studies report significant reductions in CLABSI rates and improvements in psychological well-being, others show no clear impact on rehospitalization or self-efficacy.

To improve the effectiveness of educational interventions, future research should prioritise standardised methods, validated assessment tools, and internationally recognised infection indicators. Involving patients in intervention design could further optimize outcomes and studies with a high level of evidence, such as randomized trials, should be prioritized. Additionally, cost-effectiveness analyses are essential to support the sustainable implementation of these interventions in healthcare settings.

## Supplementary Information

Supplementary Material 1.

Supplementary Material 2.

Supplementary Material 3.

## Data Availability

No datasets were generated or analysed during the current study.

## Abbreviations

CLABSI: Central line-associated bloodstream infection
CRBSI: Catheter-related bloodstream infections
CRIs: Catheter-related infections
CVCs: Central venous catheters
ICUs: Intensive care units
MMAT: Mixed Methods Appraisal Tool
PICCs: Peripherally inserted central catheters
