# Supplementary material for: Impact of educational interventions provided to patients with a central venous catheter and their informal caregivers: a systematic review
Source: Antimicrob Resist Infect Control. 2025 Jun 11;14:67. doi: 10.1186/s13756-025-01583-w (PMC12153143; doi:10.1186/s13756-025-01583-w)
Supplement: Supplementary file 1 — Supplementary Material 1. [file 13756_2025_1583_MOESM1_ESM.docx]

**Identification of studies via databases and registers**

Records identified from

Databases (n = 789):

Pubmed (n = 254)

Embase (n = 535)

Duplicate records removed

*before screening*:

(n = 246)

**Identification**

Records excluded from title / abstract

(n = 528)

*Reviews, case reports, conference abstracts*

*Written in languages other than French or English*

*Concerned staff education*

*Did not apply to central venous catheters*

*(i.e., peripheral venous catheters, urinary catheters, catheters for peritoneal dialysis).*

*Impact of education not evaluated*

Records screened

(n = 543)

**Screening**

Reports sought for retrieval

(n = 15)

Reports excluded: n = 1

*Reason: educational intervention out of scope (i.e., chronic kidney disease education programme)*

Full texts assessed for eligibility

(n = 15)

Additional records included from hand searching references (n = 6)

Studies included in review

(n = 20)

**Included**
