# Supplementary material for: Impact of educational interventions provided to patients with a central venous catheter and their informal caregivers: a systematic review
Source: Antimicrob Resist Infect Control. 2025 Jun 11;14:67. doi: 10.1186/s13756-025-01583-w (PMC12153143; doi:10.1186/s13756-025-01583-w)
Supplement: Supplementary file 2 — Supplementary Material 2. [file 13756_2025_1583_MOESM2_ESM.docx]

**Table 1 (Supplement).** Scores for assessing the quality of studies using the MMAT (Mixed Methods Appraisal Tool)

|  | **Methodological quality criteria** | | | | | **Overall score** |
| --- | --- | --- | --- | --- | --- | --- |
| **Randomized trials** | **Is randomization appropriately performed?** | **Are the groups comparable at baseline?** | **Are there complete outcome data?** | **Are outcome assessors blinded to the intervention provided?** | **Did the participants adhere to the assigned intervention?** |  |
| Emery *et al.*(35) | U | N | Y | N | Y | 2/5 (40%) |
| Li *et al.*(29) | U | Y | Y | U | Y | 3/5 (60%) |
| Liu *et al.*(26) | U | Y | Y | U | Y | 3/5 (60%) |
| Moller *et al.*(33) | Y | Y | Y | Y | Y | 5/5 (100%) |
| Raybin *et al.*(36) | U | N | Y | U | Y | 2/5 (40%) |
| Smith *et al.*(39) | U | Y | Y | Y | Y | 4/5 (80%) |
| Wang *et al.*(25) | U | Y | Y | U | Y | 3/5 (60%) |
| Average score |  |  |  |  |  | 3.1/5 (62%) |
| **Quantitative studies without randomization** | **Are the participants representative of the target population?** | **Are measurements appropriate regarding both the outcome and intervention (or exposure)?** | **Are there complete outcome data?** | **Are the confounders accounted for in the design and analysis?** | **During the study period, is the intervention administered (or exposure occurred) as intended?** |  |
| De la Maza *et al.*(43) | Y | Y | Y | N | U | 3/5 (60%) |
| Drews *et al.*(42) | U | Y | Y | N | U | 2/5 (40%) |
| Hicks *et al.*(44) | U | Y | U | Y | U | 2/5 (40%) |
| Hilberath *et al.*(38) | Y | Y | Y | N | U | 3/5 (60%) |
| Lo Vecchio *et al.*(37) | N | Y | Y | N | Y | 3/5 (60%) |
| Park *et al.*(32) | N | Y | Y | N | U | 2/5 (40%) |
| Pierick *et al.*(40) | N | U | Y | N | U | 1/5 (20%) |
| Tan *et al.*(41) | U | Y | Y | N | U | 2/5 (40%) |
| Wong *et al.*(31) | U | Y | Y | N | U | 2/5 (40%) |
| Yap *et al.*(28) | N | Y | Y | N | U | 2/5 (40%) |
| Average score |  |  |  |  |  | 2.2/5 (44%) |
| **Descriptive quantitative studies** | **Is the sampling strategy relevant to address the research question?** | **Is the sample representative of the target population?** | **Are the measurements appropriate?** | **Is the risk of nonresponse bias low?** | **Is the statistical analysis appropriate to answer the research question?** |  |
| Moller *et al.*(34) | Y | N | Y | Y | *not concerned** | 3/4 (75%) |
| Petroulias *et al.*(30) | Y | N | N | Y | N | 2/5 (40%) |
| Veyrier *et al.*(27) | Y | N | N | Y | *not concerned** | 2/4 (50%) |
| Average score |  |  |  |  |  | 2.3/5 (55%) |

*The studies were classified according to type and evaluated on five specific criteria. The overall score represents the number of criteria met out of the five assessed. "Undetermined" responses were included in the calculation of the overall score where relevant. *Criteria that were not evaluated because they were not relevant are indicated “not concerned”. For more details on the criteria assessed, please refer to the Mixed Methods Appraisal Tool (2018). Y: yes, N: no, U: undetermined*
