# Supplementary material for: Impact of educational interventions provided to patients with a central venous catheter and their informal caregivers: a systematic review
Source: Antimicrob Resist Infect Control. 2025 Jun 11;14:67. doi: 10.1186/s13756-025-01583-w (PMC12153143; doi:10.1186/s13756-025-01583-w)
Supplement: Supplementary file 3 — Supplementary Material 3. [file 13756_2025_1583_MOESM3_ESM.docx]

**Table 2 (Supplement)**

| **Author(s) (year)**  **Country**  **[Ref. No.]** | **Main outcome measure showing a *significantly* positive impact of the educational intervention.** | **Detailed characteristics of the intervention** |
| --- | --- | --- |
| De La Maza et al.(43)  Chile | **Outcome knowledge** at 1 (D1, pre-test), 10 and 90 days (D10 and D90 respectively, post-test).  D1: CG (number of correct answers) = 12+/-1 vs EG = 11 +/-2; p NS  D10: CG n=12+/-1 vs EG n= 14+/-1 p<0.0001  D90: CG n=13+/-2 vs EG n=14+/-1 p=0.01  **Outcome** **infectious complication** (see figure 3 in the article) | Nursing educators. Face-to-face individual sessions, 4 h in total over 3 consecutive days. The main CVC care measures were discussed alongside other topics, such as the pathophysiology of cancer and the side effects of chemotherapy. Caregivers received a printed copy of the content. |
| Drews et al.(42)  USA | **Outcome infectious complication** (see figure 3 in the article) | Nursing educators. Face-to-face interviews. Use of a DVD and a competency checklist of CVC care skills. Educational intervention over 4 days with 1 session/day. |
| Hicks et al.(44)  USA | **Outcome knowledge / self-management.** Pre-post difference with the Comfort Survey indicated significant increase for skill, knowledge and comfort for the pre- and the post 2 (1 month after) and 3 (2 months after) p<0.001 | Nursing educators. Collective sessions (max 6). Classes, two hours long, in a centralized location, away from the inpatient units, four times a week. Multimodal methods, including teach-back, demonstration, short videos, handouts (handbook with clear content and photos), hands-on-practice and verbal explanation. After the course, participants completed a self-assessment form so that the bedside or clinic nurse could reinforce the training in recognition of their participation in the course. |
| Hilberath et al.(38)  Germany | **Outcome infectious complication** (see figure 3 in the article) | Educational program included repetitions extending over 7–10 sessions with one session per day. Both theoretical knowledge and practical skills were taught with (hands-on) training on manikins. CVC care and complication management were discussed. Written educational materials were provided to patients and caregivers. |
| Liu et al.(26)  China | **Outcome complications (all causes)**  CG n=37 (88.10%) vs EG n=7 (16.67%) p<0.001  Infectious complication: see figure 3 in the article  Displacement: CG n=10 (83.3%) vs EG n=2 (16.67%) p=0.016 | Nursing educators. Individualized education to teach PICC maintenance and self-management techniques. Monthly meetings to watch and discuss home care videos of PICC patients. |
| Lo vecchio et al.(37)  Italy | **Outcome infectious complication** (see figure 3 in the article)  Cumulative CLABSI rate among patients whose caregivers were fully trained at the time of infection was lower (n=2, 1156 central lines days, 1.74/1000 CL-days) than those whose caregivers were not trained at the time of infection (n=13, 1066 central lines days, 12.2/1000 CL-days, P < 0.05). | In total, 9 educational sessions lasted approximately 2 months. Each session (60-90 min) was attended by 1 or 2 caregivers. The first session consisted of an overview of infection prevention measures and CVC care. The second and third sessions focused on individual and environmental hygiene measures. The remaining sessions consisted of practical experience on mannequins (3 sessions) and patients (3 sessions). At the final session, a locally produced video clip illustrating the procedures for asepsis and care of the CVC insertion site was provided to all training participants. In addition, a checklist for CVC management at home was developed and distributed to training participants |
| Moller et al.(33)  Denmark | **Outcome infectious complication** (see figure 3 in the article) | Nursing educators. Individualised patient education. Theoretical knowledge and practical application of techniques. The educational programme started 1 to 6 weeks after catheter insertion and was usually completed in 2 months. |
| Park et al.(32)  South Korea | **Outcome self-management** (questionnaire developed by the authors)  Pre-test: CG 16.17+/-2.76 vs EG 15.52 +/-2.89  Post-test: CG 19.33+/-3.93 vs EG 22.52+/-2.71  p (time effect) ≤ 0.001  p (group effect) NS  **Outcome complication (all causes)**  CG n=12 (50%) vs EG n=4 (19%)  p=0.03  Occlusion and bloodstream infections decreased between the two time periods; however, these differences were not statistically significant. | Nursing educators. Face-to-face individual sessions (didactic method) and learning of technical gestures on a model. Patients also receive a CVC management booklet. Evaluation of CVC self-management tasks using a checklist. Educational intervention every week over 6 weeks with 4 sessions of 50-min. |
| Petroulias et al.(30)  USA | **Outcome complication (occlusion)**  0 occlusion (vs 14-36% in the literature, p<0.001) | Nursing educators. Face-to-face session nurse and coaching via FaceTime for their first independent flushing procedure by means of an electronic tablet (given to each participant for 2 weeks). Access to a video that outlined the 10 steps of flushing. |
| Pierick et al.(40)  Canada | **Outcome complication (all causes)**  Before intervention 7.88 complications per 1000 catheter days  vs after intervention 2.65 per 1000 catheter days  p 0.046  All three types of CVC-related complications (line breakages, occlusion, and bloodstream infections) decreased between the two time periods; however, these differences were not statistically significant. | Nursing educators. Individual training program with printed materials (manual). Families and caregivers were involved in the production of 3-to-5 minute educational videos: videos for handwashing, dressing changes, hooking up to PN, unhooking from PN, and flushing of the CVC. |
| Smith et al.(39)  USA | **Outcome anxiety and depression (evaluated clinically and through a questionnaire)**  At 6 months  CG 17/38 (45%) vs EG 14/35 (40%) p 0.03  At 18 months  CG 21/38 (55%) vs EG 18/35 (51%) p NS  **Outcome rehospitalization**  Fewer rehospitalizations due to infection (p = 0.017). No significant difference for other causes  **Outcome infectious complication** (see figure 3 in the article) | Nursing educators. Each educational intervention packet included a videotape (12 to 15 minutes) with accompanying pamphlet, a self-monitoring check-list, and a diary. Research nurses instructed patients to write in the diaries 3 times weekly for 12 weeks on topics related to infection, depression, and other HPN daily care management problems and to report and discuss these with their attending physician. The videotaped intervention included (1) infection prevention by self-monitoring, (2) therapeutic writing and short massages for decreasing reactive depression, and (3) problem-solving partnerships with professionals. |
| Tan et al.(41)  Taiwan | **Outcome satisfaction**  Survey (12 items, scored using a Likert-type scale)  CG 50.37 ± 6.3 vs EG 57.13 ± 4.4  p < 0.05 | Face-to-face individual training sessions (20 min). Patients received a health instructional handbook, a 10-min instructional video corresponding with the contents of the handbook, and a checklist of CLABSI prevention guidelines. |
| Wang et al.(25)  China | **Outcome anxiety and depression** The SAS (Anxiety Self-assessment Scale) and SDS (Depression Self-assessment Scale) scale scores were decreased in both groups after the intervention, and the intervention group showed lower scores than the control group (*p*<0.001).  **Outcome complication (all causes)**  CG n=16 (26.67%) vs EG n=6 (10%)  p 0.036  **Outcome self-management**  CPPSM (Cancer Patients PICC Self-management Scale) score increased after the intervention, and the scale’s score in the intervention group was higher than in the control group (p<0.05) | Previous patients who had been successfully treated were invited to give lectures. The placement process and the advantages of PICC, its role, and the key points during placement were explained using drawings, pictures, and other visual methods. Patient education cards and brochures were distributed to explain the precautions to be taken after placement. The risk factors for complications during placement and the prevention of complications were explained to the patients using patient education through WeChat groups and telephone follow-up. Educational videos on the maintenance of PICCs and checklists were used to supervise the patients and their families to independently carry out daily catheter maintenance. |
| Yap *et al.*(28)  Australia | **Outcome complication (all causes)**  Before intervention (historical cohort) n=11/27 (40.7%)  vs after intervention n=14/88 (15.9%) p 0.006  No significant difference for infectious complications alone. | Nursing educators. No information on the teaching tools used. All patients were made aware of the importance of hygiene and were taught to consult a nurse or doctor in the event of problems such as pain, swelling, or erythema. |

CG: control group; EG: experimental group; NS: not significant
